# New Haven Dental Clinic

**Published:** 1904-08

**Authors:** 


### Domestic Correspondence.

### NEW HAVEN DENTAL CLINIC.

[The following account of the recently organized Dental Clinic at New Haven, Conn., will be read with interest. While it is not the first attempt of the kind to render service to the poor, it has features superior to other attempts preceding it.—Ed.]

"The latest addition to the New Haven Free Dispensary, and one of the most important, is the dental clinic which is now in operation under the direction of the New Haven Dental Association. The dispensary is, of course, under the direction and management of the Yale Medical School, but the physicians and students who have been doing work there have not been able to do any dental work, and need for a department of this kind has been felt for some time. During the winter it was proposed to the New Haven Dental Association that a clinic of this sort be established under its direction. The matter was brought before the Association and was almost unanimously decided upon. Dr. William H. Carmalt, the director of the surgical clinic at the dispensary, immediately appointed Dr. E. S. Gaylord, Vice President of the Dental Association, director of this new clinic. It was through his efforts and those of Dr. George E. Nettleton that the room has been furnished and completed for operation.

"The room itself, with its tile floor, white painted walls, and white enamelled furniture, is an example of cleanliness and purity. The furniture was especially made for this purpose, and a hose might be turned into the room without injury. The furniture consists of two operating-chairs, the upholstery of which is dark red leather, two ordinary metal chairs, a metal chest of drawers, and a metal and glass case for instruments; also two electrical engines. An interesting feature of the furnishing of the room is the fact that it was donated to the society by Mr. Clarence R. Hooker, Mr. Donald R. Hooker, and Miss Elizabeth R. Hooker, the children of the late Frank H. Hooker of this city. It was a purely unsolicited donation, and for that reason all the more valuable. The furnishings of the room are practically the property of the dispensary, whose duty it is to care for them, furnish new pieces if necessary, and replace lost or broken articles.

"This is to all practical purposes the first clinic of its kind in New England. It furnishes the dental profession of this city an opportunity to do charitable work for the worthy poor who are unable to pay for such services. And only that class of poor who can prove that they are unable to pay for this work are admitted. The fact that this clinic was needed has been proved by the number of people who have availed themselves of this opportunity up to the present time. The room is open two afternoons a week,—namely, Tuesday and Thursday,—from two until four o'clock, or until all who apply for help have been cared for. There are two operators in attendance each afternoon, who have been appointed by the director. Dr. Gaylord has made out the appointments for six months, giving each dentist four afternoons in that time. It has been so arranged that two strangers will not be there the same afternoon, that is, when a man goes for the first time, there will also be an operator in attendance who has been there before, and who can instruct the new man to a certain extent. If the number of applicants increases, so that the two afternoons will not be sufficient, additional time will be given and other operators assigned. The members of the dental society have been very enthusiastic about the work and very ready and willing to do their part towards carrying out the plans. It is to be hoped that this enthusiasm will not decrease, as it is a good and important work, and one that ought to be carried out.

"The card system is used in keeping record of the work done, and a complete record of each case is kept, including name, age, and address of the patient, date of operation, character of operation, and name of operator. Although the work has only been going on about three weeks, it has been running along very smoothly, and everything promises a very successful future."—*Saturday Chronicle,* New Haven.

